# 8.J. Workshop: Transfer and adoption of digitally enabled integrated care good practices: the JADECARE Joint Action

**DOI:** 10.1093/eurpub/ckac129.506

**Published:** 2022-10-25

**Authors:** Organised by: Kronikgune Institute for Health Services Research (Spain), Chair persons: Jon Txarramendieta (Spain)

## Abstract

The ageing population and the burden of chronic conditions is increasing the demand for more comprehensive, efficient and smarter healthcare. The EU funded Joint Action JADECARE (Joint Action on implementation of Digitally Enabled integrated person-centred CARE) intends to reinforce the capacity of health authorities to successfully address the health system transition to digitally enabled integrated person-centred care. JADECARE involves 45 organisations from 16 different EU countries covering different funding systems (both Bismarck, Beveridge and mixed) holding different levels of penetration, adoption and maturity of integrated care. It focuses on the transfer and adoption of four original Good Practices (oGPs) to the contexts of 21 Next Adopters (NA). The project started on October 1 2020 and ends on September 30 2023. The oGPs that are being transferred in JADECARE concern integrated care, chronic conditions, self-care, prevention and population health. They are: The Basque Health strategy in ageing and chronicity: integrated care (Spain), the Catalan open innovation hub on ICT-supported integrated care services for chronic patients (Spain), the OptiMedis Model-Population-based integrated care (Germany) and the Digital roadmap towards an integrated health care sector (Denmark). Due to the heterogeneity of the NA, the transfer strategy of JADECARE enables them to customize the parts of the oGPs that will be transferred and adapted to its local context, considering its needs, local strategies and implementation goals. The JADECARE three-step implementation strategy has been designed to be appropriate from the scientific point of view, applicable considering data availability and feasible according to project's timeline and resources:

• Pre-implementation: definition of Local interventions and action plans.

• Implementation: roll-out and operation, based on two Plan-Do-Study-Act cycles.

• Post-implementation: result analysis, impact assessment and reporting on learnings.

By the end of 2022, the implementation phase will be completed, and all NA will start analyzing their results and reporting on learnings. Currently, NAs have defined their interventions and action plans for implementation and are finalizing the first of the two PDSA cycles that they will be performing within JADECARE. 3 of the NAs are implementing interventions at national level, 14 at regional level and 4 at local level. Most of the NAs implement interventions related to digitally enabled integrated care (36), 11 are related to risk stratification and 9 to patient empowerment. This workshop explores the key elements and challenges to be addressed when transferring Good Practices (GP) to heterogeneous health systems with different needs, aims and scopes. It will also highlight the experience of two NA: Marche region, in Italy and Olomouc, in Czech Republic and promote the discussion among speakers and attendees to highlight the challenges and key elements to enable the transfer of GPs.

**Key messages:**

• JADECARE will impact on EU health systems by implementing innovative solutions and helping to change the model of care provision. It will also settle the basis to implement them at large scale.

• The long-term effect of JADECARE is supported by involving stakeholders at regional and national levels to provide political support and commitment to sustained integrated care.

